# Curcumin-Loaded Lipid Nanoparticles: A Promising Antimicrobial Strategy Against *Enterococcus faecalis* in Endodontic Infections

**DOI:** 10.3390/pharmaceutics17010108

**Published:** 2025-01-14

**Authors:** Sónia Ferreira, Liliana Grenho, Maria Helena Fernandes, Sofia A. Costa Lima

**Affiliations:** 1 University Institute of Health Sciences (IUCS), Cooperative CESPU, CRL, 4585-116 Gandra, Portugal; sonia.ferreira@iucs.cespu.pt; 2 BoneLab—Laboratory for Bone Metabolism and Regeneration, Faculdade de Medicina Dentária, Universidade do Porto, Rua Dr. Manuel Pereira da Silva, 4200-393 Porto, Portugalmhfernandes@fmd.up.pt (M.H.F.); 3 Laboratório Associado para a Química Verde-Rede de Química e Tecnologia (LAQV, REQUIMTE), Faculdade de Medicina Dentária, Universidade do Porto, Rua Dr. Manuel Pereira da Silva, 4200-393 Porto, Portugal; 4 Laboratório Associado para a Química Verde-Rede de Química e Tecnologia (LAQV, REQUIMTE), Instituto de Ciências Biomédicas de Abel Salazar, Universidade do Porto, Rua de Jorge Viterbo Ferreira 228, 4050-313 Porto, Portugal

**Keywords:** antibacterial activity, cytocompatibility, curcumin, endodontic treatment, *Enterococcus faecalis*, lipid nanoparticles

## Abstract

**Background/Objectives:** This study aims to evaluate the efficacy of curcumin (CUR), a natural polyphenol with potent antimicrobial and anti-inflammatory properties, when formulated as solid lipid nanoparticles (CUR-loaded SLN) against *Enterococcus faecalis*. **Methods:** Solid lipid nanoparticles (SLNs) were prepared as a carrier for CUR, which significantly improved its solubility. SLNs made with cetyl palmitate and Tween 80 were obtained via the hot ultrasonication method. The physicochemical properties of CUR-loaded SLNs were evaluated, including their size, stability, and release profile. Antimicrobial testing was conducted against both sessile and planktonic *E. faecalis* populations. Cytotoxicity was assessed on human gingival fibroblasts. **Results:** The CUR-loaded SLNs exhibited about 200 nm and a −25 mV surface potential, and the encapsulation of CUR did not affect the physicochemical properties of SLNs. CURs were released from SLNs in a controlled and sustained manner over 100 h. The nanoparticles remained stable for at least two months when stored at 4 °C or 25 °C, making them suitable for clinical use. Antioxidant activity was confirmed through DPPH and ABTS assays. Free CUR significantly reduced the planktonic *E. faecalis* CFU counts by approximately 65% after 24 h of exposure. However, this inhibitory effect diminished with longer exposure times (48 and 72 h). Antimicrobial activity studies of CUR-loaded SLNs showed dose- and time-dependent effects, in the 2.5–10 µg/mL range, against both sessile and planktonic *E. faecalis* populations, over 24 to 72 h. The CUR-loaded SLNs showed good cytocompatibility with human fibroblasts up to 2.5 μg/mL, suggesting low toxicity. **Conclusions:** CUR-loaded SLNs demonstrate significant antimicrobial activity against *E. faecalis*, along with good cytocompatibility, indicating their potential as an effective adjunct therapy in endodontic treatments.

## 1. Introduction

Endodontic treatment is a dental practice designed to treat diseases or injuries affecting the dental pulp and surrounding tissues. The primary objective of this root canal procedure is to remove or significantly reduce microorganisms and their derivatives, ensuring the disinfected tooth remains protected against microbial intrusion [1]. The intricate nature of the root canal system represents a challenge for the cleaning and disinfection process. Root canal shaping involves the use of various manual and rotary instruments. However, these tools mainly focus on the central area of the root canal, potentially leaving structural irregularities filled with debris, necrotic pulp residues, microorganisms, and their derivatives. This may result in apical periodontitis [2,3]. Whatever the canal preparation technique applied, about 35% of the root canal surface continues untouched [4]. Calcium hydroxide remains the most used intracanal dressing due to its antimicrobial properties, inhibition of osteoclast activity, promotion of mineralization, and tissue dissolution [5,6]. Its antimicrobial action stems from hydroxyl ions that create an alkaline pH unsuitable for microbial survival [7]. However, its low aqueous solubility, slow diffusion, and buffering by dentin proteins limit its ability to rapidly increase pH and effectively eliminate microorganisms, particularly in the apical third of the root canal [8,9,10,11,12].

Curcuminoids are natural polyphenolic compounds derived from the rhizome of the turmeric plant, scientifically known as *Curcuma longa*, a member of the Zingiberaceae family. These compounds exhibit diverse biological functions, primarily due to their antioxidant properties [13]. Curcumin (CUR), a particular curcuminoid, has shown in vitro antimicrobial activity against various microorganisms, namely fungi [14,15] and bacteria [16,17,18]. Recent studies have shown that CUR can effectively inhibit the tooth surface attachment of *Streptococcus mutans* [19]. Studies have demonstrated a synergistic effect that CUR presents in combination with various antibiotics towards *Staphylococcus aureus* [20]. Curcumin has been considered a promising tool for inclusion in combined treatments against pathogens in humans due to its availability, high efficacy, and low cytotoxicity [21]. Curcumin disrupts bacterial cytokinesis by inducing filamentation, preventing the formation of the cytokinetic Z ring, and inhibiting bacterial proliferation. Despite its remarkable pharmacological advantages, researchers continue to struggle with challenges related to CUR’s poor solubility in water, low oral bioavailability, chemical instability, limited absorption, rapid metabolism, and elimination [22]. The application of nanotechnology-based delivery systems allows for improving CUR solubility problems and facilitates a sustained release [18,23]. The ability of nanoparticles to attach to the surfaces of bacterial cells is contingent upon their surface chemistry, charge, and hydrophobicity. Additionally, they may penetrate the pores in the cell membrane, where they can disrupt the synthesis of bacterial proteins and DNA. This impairment of the bacterial function leads to damage to the bacteria [24]. Recent studies in endodontic treatments indicate that polymeric nanoparticles of poly (lactic-co-glycolic acid) (PLGA) loaded with CUR hold promise as an adjuvant therapy for endodontic infections. At a concentration of 325 μg/mL, photoactivated CUR-loaded PLGA nanoparticles demonstrated the most significant reduction in the viability of endodontic bacteria. Regarding biocompatibility, both CUR-loaded PLGA nanoparticles and empty nanoparticles showed cell viability above 80% [25]. Additionally, the conjugation of CUR-loaded nanoparticles with other photosensitizers like indocyanine green and metformin may improve the anti-biofilm activity of photodynamic therapy (PDT) towards *E. faecalis* [26]. Despite the investigations into the combined action of CUR with PDT, the results show that the CUR-loaded nanoparticles achieve the minimum inhibitory concentration against *E. faecalis* with a lower CUR dose compared to its free form [25]. CUR-loaded nanoparticles can also be used as a sonosensitizer. Sonodynamic antimicrobial chemotherapy, when combined with ultrasound waves, appears to enhance therapeutic efficacy, likely due to an increased production of reactive oxygen species [27]. Lipid-based nano-delivery systems have good affinity to lipophilic compounds like CUR and, so far, have been less studied than polymeric nanoparticles for endodontic treatment [28]. Lipid nanoparticles offer unique advantages over other nanocarriers (e.g., polymeric nanoparticles and inorganic nanoparticles) in endodontic applications due to the following: (i) their biocompatibility and biodegradability that minimize the risk of adverse reactions and cytotoxicity in the sensitive oral environment; (ii) their enhanced drug loading as lipid nanoparticles provide excellent encapsulation efficiency for both hydrophilic and lipophilic drugs; (iii) their improved stability in relation to liposomes, as they are more stable in physiological conditions and during storage with a longer shelf life; (iv) their biofilm penetration as lipid nanoparticles can overcome the dense extracellular matrix of microbial biofilms commonly found in infected root canals, allowing antimicrobial agents to reach and act on embedded bacteria effectively; (v) cost-effective since the production of these nanoparticles is relatively low cost and scalable, making them practical for clinical and commercial applications compared to certain polymer-based systems or advanced hybrid nanocarriers and potential to enhance the stability and bioavailability of therapeutic compounds such as CUR. These features make lipid nanoparticles particularly suited for addressing the challenges of endodontic infections and ensuring effective intracanal delivery of therapeutic agents [29,30,31].

In sum, CUR has gained significant attention in the medical and dental fields for its diverse biological properties [31,32]. Its potential as an adjunct in clinical endodontic therapy is underscored by the interplay between its biological effects and therapeutic applications. For example, CUR’s anti-inflammatory properties manage periapical inflammation, reducing pain, and promoting healing in conditions like apical periodontitis or post-treatment flare-ups, while its antioxidant properties help mitigate oxidative stress associated with tissue damage in inflammatory conditions, facilitating the healing of periapical lesions and minimizing collateral damage to host tissues [33]. Overall, the potential applications in endodontic treatments include adjunct in root canal irrigation to enhance antimicrobial action; component in intracanal formulations to reduce inflammation and support healing; and, even, as an agent in photodynamic therapy for effective microbial elimination. Considering the physicochemical properties of CUR, enhancing its efficacy, water solubility, and safety through advanced formulations, and thorough clinical validation is essential. This need prompted the design of novel solid lipid nanoparticles for CUR delivery, which have been less explored than polymeric nanoparticles in the context of endodontic treatment, the focus of this study. Solid lipid nanoparticles (SLNs) consist of a solid lipid matrix that provides a stable structure for encapsulating and delivering bioactive compounds. SLNs offer biocompatibility, physical stability, high drug loading capacity, protection of active ingredients, improved bioavailability, and are relatively easy to produce [34,35].

This study aimed to evaluate the potential of CUR-loaded solid lipid nanoparticles (CUR-loaded SLNs) as a novel adjunct therapy for controlling *E. faecalis* biofilms in endodontic infections. By leveraging nanotechnology, this research intends to offer a promising alternative to traditional antimicrobial agents, advancing more effective endodontic treatments while helping to prevent the development of antimicrobial resistance.

## 2. Materials and Methods

### 2.1. Materials

Tween^®^ 80, dimethyl sulfoxide (DMSO), Dulbecco’s phosphate-buffered saline (DPBS) (10×) and curcumin (CUR) (Sigma-Aldrich, St. Louis, MO, USA), absolute ethanol (Thermo Fisher Scientific, Waltham, MA, USA), cetyl palmitate (Gattefossé, Neuilly-Sur-Seine, France), Amicon^®^ Ultra Centrifugal Filters (Ultracel-50 KDa, Darmstadt, Germany) were obtained from Merck Millipore (Wicklow, Ireland). *Enterococcus faecalis* ATCC 29212 was selected as the test strain, and reagents included Tryptic Soy Broth (TSB) and Tryptic Soy Agar (TSA) (Liofilchem, Roseto degli Abruzzi, TE, Italy), and Resazurin salt (Sigma-Aldrich, St. Louis, MO, USA). Human dermal gingival fibroblasts (AG09319) were sourced from the Coriell Institute (Camden, NJ, USA), while alpha-minimum essential medium (α-MEM), fetal bovine serum (FBS), penicillin, streptomycin, amphotericin B, TrypLE™ Express Enzyme, and phosphate-buffered saline (PBS) (Gibco, Waltham, MA, USA), 3-(4,5-Dimethylthiazol-2-yl)-2,5-diphenyltetrazolium bromide (MTT), formaldehyde, Triton X-100, and bovine serum albumin (BSA) (Sigma-Aldrich, St. Louis, MO, USA), Flash Phalloidin^TM^ Green 488, Hoechst 33342, and Calcein-AM (BioLegend, San Diego, CA, USA), and propidium iodide (PI) were also obtained (BD Biosciences, Franklin Lakes, NJ, USA).

### 2.2. Methods

#### 2.2.1. Preparation of Solid Lipid Nanoparticles

Curcumin-loaded solid lipid nanoparticles (CUR-loaded SLNs) and empty SLNs were prepared using the hot ultrasonication method [36]. The solid lipid of cetyl palmitate (150 mg), the stabilizing agent non-ionic surfactant Tween 80^®^ (47 mg), and the bioactive molecule CUR (1 mg) were heated to 65 °C in a water bath. Upon melting this mixture, 7 mL of pre-warmed water was added. To control size and size distribution, the suspension was homogenized with a probe-sonicator (VCX130, Sonics & Materials, 115 Newtown, CT, USA) at an amplitude frequency of 70% for 5 min. Unloaded SLNs were prepared following the same procedure but without the addition of CUR.

#### 2.2.2. Characterization of Solid Lipid Nanoparticles

CUR-loaded SLNs and unloaded SLNs were analyzed in terms of mean size, polydispersity index (PDI), and zeta potential using a ZetaPALS zeta potential analyzer (Brookhaven Instruments Corporation; Holtsville, NY, USA). For the size determination, each sample was diluted in double-deionized water at a 1:200 ratio. The measurements were conducted at 20 °C, with six runs lasting 2 min each. For the zeta potential determination, the setup of the Electrophoretic Light Scattering included an electrode operating at a 90° scattering angle at 20 °C. Each measurement consisted of six runs with 10 cycles per run.

For the quantification of CUR’s entrapment efficiency (EE), the CUR-loaded SLNs were diluted 66-fold in double-deionized water and separated using Amicon^®^ filter units [35]. The non-encapsulated CUR, present in the supernatant, was quantified by UV–vis spectrophotometry (Jasco V-660 Spectrophotometer, Jasco Corporation, Easton, MD, USA) at 435 nm. A calibration curve was made with CUR standards in 70% ethanol, ranging from 0.6 to 20 μg/mL, yielding the correlation OD435 = 0.073[CUR] + 0.064 (r^2^ = 0.9989). The EE was determined using the following equation:

EE (%) = (Total initial drug − Drug in the supernatant)/Total initial drug × 100

#### 2.2.3. Storage Stability

Evaluation of the storage stability of CUR-loaded SLNs was studied at 4 °C and 25 °C. The formulation was kept in sealed glass vials protected from light. To determine the nanoparticle’s stability measurements of size, the PDI, zeta potential, and drug content were examined over 8 weeks.

#### 2.2.4. In Vitro Curcumin Release Kinetics

The in vitro release profile of CUR from the SLNs was achieved using the dialysis bag method. Thus, 2 mL of either free CUR (0.85 mg/mL in oleic acid) or CUR-loaded SLNs was transferred into the dialysis bag prior to submersion in a beaker containing 80 mL of release medium (PBS with 10% ethanol) for 72 h. Samples of 1 mL were collected and replaced with an equal volume of fresh release medium. CUR quantification followed the description in Section 2.2.2. To understand the release mechanism, mathematical models were fitted to the experimental data (Table 1). The best-fit model was selected based on the regression coefficient (r^2^) value.

#### 2.2.5. Antibacterial Activity of SLNs and CUR-Loaded SLNs

##### Planktonic Growth Inhibition Assay

The antibacterial activity of SLNs and CUR-loaded SLNs against *E. faecalis* planktonic growth was evaluated using the colony-forming unit (CFU) counting method. The assay was carried out in 96-well plates, and a bacterial suspension of 10^6^ CFU/mL in TSB was transferred to each well. SLNs or CUR-loaded SLNs were added to achieve final concentrations of 2.5, 5, and 10 µg/mL in CUR. Control wells were prepared likewise without SLN formulations. The plates were incubated for up to 72 h at 37 °C with constant shaking at 150 rpm. At each specific time point (24, 48 and 72 h), samples were serially diluted and plated on TSA plates. Following a 24 h incubation period at 37 °C, the CFUs were counted. The results are expressed as a percentage of cultivable bacteria relative to the control group, which was set at 100%. The antibacterial activity of free CUR was evaluated with planktonic *E. faecalis*, following the experimental setup described above for CUR-loaded SLNs.

##### Antibiofilm Formation Assay

The antibacterial activity of SLNs and CUR-loaded SLNs against *E. faecalis* biofilm formation was evaluated using the resazurin assay. Each well of 96-well plates was inoculated with a bacterial suspension of 10^8^ CFU/mL prepared in TSB and incubated at 37 °C and 150 rpm for 2 h to allow for bacterial adherence. Following the initial incubation period, the wells were gently washed with 0.9% NaCl solution to remove any planktonic and loosely attached cells. Subsequently, varying concentrations of SLNs or CUR-loaded SLNs (2.5, 5, and 10 µg/mL) were added, and the plates were incubated at 37 °C and 150 rpm for 24, 48, and 72 h. The control wells were inoculated with the bacterial suspension and TSB without SLN formulations. At each time point, the wells were rinsed with 0.9% NaCl, and TSB containing 10% resazurin (0.1 mg/mL) was added. Upon color change, fluorescence intensity (excitation: 530 nm; emission: 590 nm) was measured using a microplate reader (Synergy HT, BioTek, BioTek^®^ Instruments Inc., Winooski, VT, USA). Results are expressed as a percentage relative to the control group, which was set at 100%.

#### 2.2.6. In Vitro Cytocompatibility

Human gingival fibroblasts were cultured at 37 °C in α-MEM complemented with FBS (10%), penicillin (100 IU/mL), streptomycin (100 µg/mL), and amphotericin B (0.25 µg/mL), in a humidified atmosphere containing 5% CO_2_. Once the cells had reached 80% confluence, they were enzymatically detached using TrypLE™ Express Enzyme and seeded at a density of 10^5^ cells/mL in 96-well microplates. After 24 h of incubation to allow for cell adhesion, the cells were exposed to SLNs and CUR-loaded SLNs, at 2.5, 5, and 10 µg/mL. Cells incubated with culture medium alone, without SLN formulations, served as the control group. The cellular response was then evaluated using the following methods.

##### Cell Viability

Cell viability was assessed using live/dead cell staining at specific time points of exposure (1, 2, and 3 days) for both empty SLNs and CUR-loaded SLNs. This assay employs fluorescent dyes, specifically calcein and propidium iodide, to label live and dead cells simultaneously, respectively. Calcein AM is a non-fluorescent hydrophobic molecule that can easily penetrate the intact membranes of live cells. Once inside the cell, intracellular esterases convert the molecule into a hydrophilic, highly fluorescent product (calcein, green), which is retained within the cytoplasm. In contrast, propidium iodide is a membrane-impermeable stain. It can only enter the cell and bind to DNA when cellular plasma membranes are damaged. At each time point, cells were incubated with Calcein AM (1 μM) and propidium iodide (as supplied) for 30 min at 37 °C, protected from light. Fluorescence was observed using a Celena S digital imaging system (Logos Biosystems, Anyang-si, Republic of Korea). Images from each experimental condition were analyzed using ImageJ (version 1.8, NIH, Bethesda, MD, USA) to quantify the number of live cells. Data are expressed as a percentage relative to the control group, which was set at 100%.

##### Cell Metabolic Activity

Cell metabolic activity was evaluated using the MTT assay following 24 h of exposure to SLNs and CUR-loaded SLNs. Cells were incubated with 10% MTT solution (5 mg/mL) for 3 h at 37 °C. Metabolically active cells reduced MTT to violet-blue formazan crystals. These crystals were subsequently solubilized in DMSO for 15 min. The absorbance of the resulting solution was measured at 550 nm using a microplate reader (Synergy HT, Biotek). Results are expressed as a percentage relative to the control group, which was set at 100%.

##### Cell Morphology

Immunofluorescence staining of the filamentous actin (F-actin) cytoskeleton was performed to evaluate cell morphology following 24 h of exposure to SLNs and CUR-loaded SLNs. Cells were initially washed with PBS and fixed with 3.7% formaldehyde for 10 min. The fixed cells were then permeabilized with 0.1% Triton X-100 for 15 min, followed by 30 min in PBS containing 1% BSA. The F-actin of the cytoskeleton was stained with Flash Phalloidin^TM^ Green 488 (1:100) for 30 min, and the nuclei with Hoechst 33342 (10 µg/mL) for 15 min. Images were captured using the Celena S Digital Imaging System (Logos Biosystems, Anyang-si, Republic of Korea).

### 2.3. Statistical Analysis

Biological studies were performed with at least three biological replications, with three technical replicas. IBM^®^ SPSS^®^ Statistics software (v. 28.0.0.0, SPSS Inc., Chicago, IL, USA) was employed for the statistical analyses. The Student’s paired *t*-test and the one-way analysis of variance (ANOVA) with post hoc Tukey HSD were employed to assess the data. The level of significance was set at *p* < 0.05.

## 3. Results and Discussion

This study produced and characterized lipid nanoparticles to improve physicochemical stability and enhance the local bioavailability of CUR for root canal therapy. Cetyl palmitate, chosen for its biocompatibility, cost-effectiveness, and established application in the pharmaceutical, cosmetic, and food industries, served as the solid lipid in the delivery system. To stabilize the SLNs, Tween^®^ 80 was selected as it acts by reducing the surface tension between the lipid and aqueous phases. This prevents the nanoparticles from aggregating, ensuring uniform and stable dispersion. Also, lipid core stabilization ensures a high drug load and prevents premature drug release.

### 3.1. Characterization of the Lipid Nanoparticles Containing Curcumin

The data obtained for the size, polydispersity index, zeta potential, and encapsulation efficiency (EE) for SLN and CUR-loaded SLN formulations are presented in Table 2. The mean particle sizes for SLN and CUR-loaded SLNs were determined to be 207 ± 6 nm and 209 ± 6 nm, respectively. This indicates that the inclusion of the bioactive molecule did not alter the size of the formulations. Currently, no indication suggests that nanoparticle size affects the efficacy of endodontic therapies [37]. Yet, the suspension of nanoparticles should be easily handled using a syringe equipped with a 30 G/25 mm needle, commonly used in dental applications. The PDI serves to represent the width of the nanoparticle size distribution. In this study, all SLNs exhibited PDIs below 0.17, indicating that the formulated nanoparticles can be considered uniform, with low size variability among the population [38]. The zeta potential value allows for the assessment of the colloidal stability of the nanoparticles. A zeta potential value of ±30 mV is necessary to ensure a stable system [38], while a neutral surface potential value indicates a propensity for nanoparticle aggregation, attributed to a reduction in repulsion forces [39]. In this study, there is no correlation between loading nanoparticles with CUR and the surface potential. The zeta potential values determined for unloaded and CUR-loaded SLNs were approximately −25 mV.

The CUR encapsulation efficiency in the cetyl palmitate/Tween 80-based SLNs was found to be high (ca. 85%), resulting in a 200-fold enhancement in its water solubility (0.6 µg/mL [40]). This can be attributed to the substantial lipophilicity of the drug being incorporated. The observed value also suggests that the lipid composition of the formulations is adequate for the loading and delivery of CUR. By encapsulating CUR within SLNs, the hydrophobic drug is enclosed in a lipid matrix, which improves its dispersion and stability in water. Moreover, the lipid core of SLNs acts as a reservoir, protecting CUR from rapid degradation while promoting its solubilization in aqueous environments.

**Figure 1:**
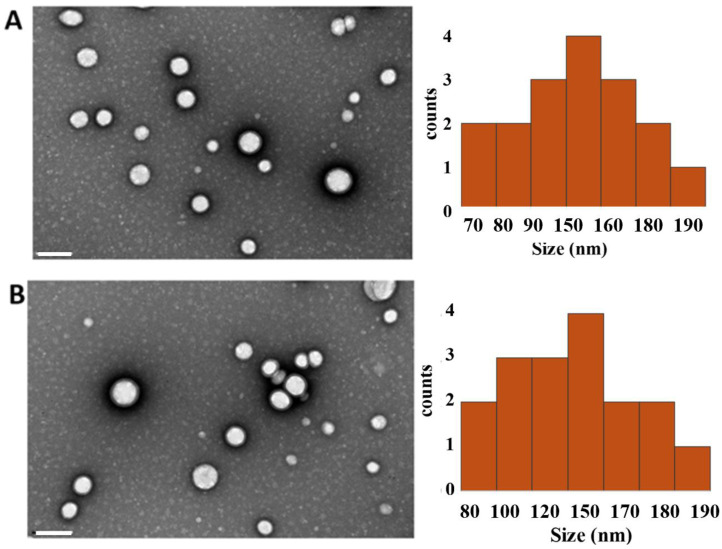
Morphology analysis. Transmission electron images (**left**) and related histograms (**right**) of (**A**) SLNs and (**B**) CUR-loaded SLNs. Scale bar 200 nm.

The morphology of SLNs was observed using TEM, as illustrated in Figure 1. The images revealed that all formulations maintained a spherical shape and that the loading of CUR did not alter their main properties. It is noted that the TEM images displayed a smaller diameter for SLNs compared to the measurement obtained through DLS (Table 2). This discrepancy arises from the differences between both techniques, as DLS measurements are based on a nanoparticle’s Brownian motion, whereas TEM captures static nanoparticles, resulting in a smaller size value.

**Figure 2:**
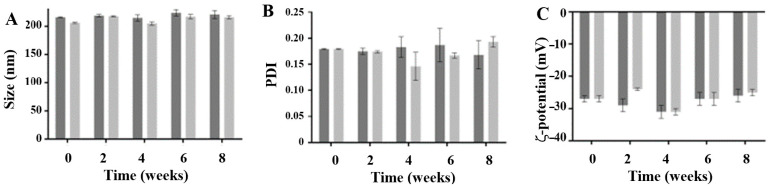
Evaluation of storage stability at room temperature (dark grey) and at 4 °C (light grey). The size (**A**), polydispersity index values (**B**) and ζ-potential (**C**) were determined over 8 weeks.

The storage stability, both at 4 °C and at 25 °C, of the CUR-loaded SLNs was assessed for 2 months. The lipid nanoparticles were stable when stored at 25 °C, as the size, PDI and zeta potential values remained similar to freshly prepared formulations during the whole assessed storage period (Figure 2). The storage temperature within the 8 weeks of the study did not influence the physiochemical stability of the CUR-loaded SLNs, and less than 10% of the entrapped bioactive compound was lost over the storage period. The stability of nanoparticles during storage is essential for their use in dentistry clinical applications. Maintaining stability over extended periods under various storage conditions ensures their effectiveness and safety in dental treatments.

**Figure 3:**
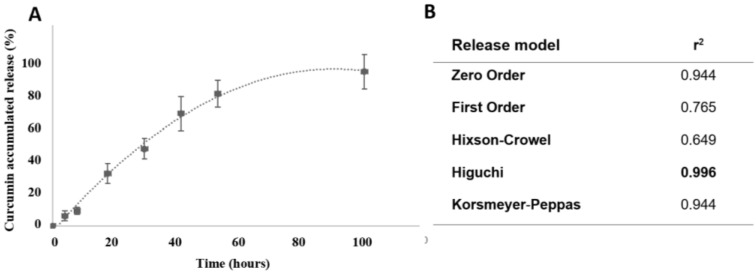
In vitro CUR release profile from the SLNs. (**A**) Cumulative release profiles of CUR from SLNs in release medium. Data are expressed as the means ± standard errors of three independent experiments (*n* = 3). (**B**) Release kinetic parameters (r^2^) for CUR-loaded SLN data obtained using several mathematical models.

The in vitro release of CUR from SLNs was evaluated in phosphate buffer with 10% ethanol at physiological pH conditions to simulate the contact of the nanoparticles following endodontic application. Figure 3A shows a slow CUR release profile from SLNs. Of notice, within the first 4 h, only 10% of drug was released from the SLNs, and upon 12 h, only 20%. These results point out that the majority of the drug (80% or more) remained entrapped within SLNs after exposure to in vitro mimetic conditions and could be slowly released in the infected tissues for up to 4–5 days. This reinforces the suitability of SLNs for the endodontic delivery of CUR, offering protection against degradation and ultimately enhancing drug bioavailability. In terms of sustained release, it has been previously demonstrated that lipids with longer carbon chains, such as cetyl palmitate, result in a prolonged release in relation to lipids with shorter carbon chains [41]. This implies that lipids with longer carbon chains possess a greater ability to encapsulate CUR within the particle core due to their lipophilic nature.

The release profile of CUR-loaded SLNs was characterized by analyzing the regression coefficients (r^2^) obtained after fitting into the various mathematical release kinetics models, in Figure 3B [42]. Based on the results, the Higuchi model was identified as the most suitable model, showing the highest r^2^ value (0.996). This mathematical model, derived from Fick’s law of diffusion, indicates a direct relationship between drug release from the matrix and the square root of time. Therefore, the release of CUR from SLNs occurs through a diffusion-controlled process, which is governed by the structure and properties of the lipid matrix. The lipid core of the nanoparticles allows for a controlled and sustained release over an extended period, crucial for maintaining therapeutic concentrations at the site of infection. This is particularly advantageous in endodontic treatments, where a long-lasting antimicrobial effect is required to prevent re-infection and enhance healing. Furthermore, the controlled release mechanism of SLNs allows for the gradual delivery of CUR, reducing the risk of drug accumulation or toxicity while enhancing its therapeutic efficacy. By sustaining an optimal concentration of CUR in the root canal, SLNs minimize the need for frequent reapplications, thereby improving patient compliance and simplifying the treatment process.

**Figure 4:**
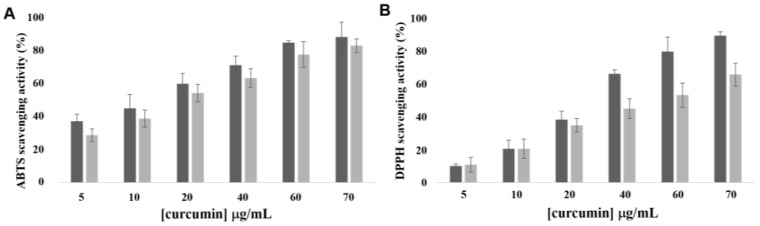
Antioxidant activity of CUR. (**A**) ABTS scavenging activity of CUR (dark grey bars) and CUR-loaded SLNs (light grey bars); (**B**) DPPH scavenging activity of CUR (dark grey bars) and CUR-loaded SLNs (light grey bars). Results are expressed as the means ± standard errors of three independent experiments (*n* = 3).

Curcumin has been widely studied for its relevant pharmacological properties, particularly its antioxidant effect, through the formation of phenoxy groups to remove free radicals [43]. Hence, to assess how encapsulation in lipid nanoparticles affects CUR’s antioxidant capacity, two scavenging assays, ABTS and DDPH, were performed (Figure 4A and Figure 4B, respectively).

The scavenging activity of CUR-loaded nanoparticles as well as free CUR is concentration-dependent in both ABTS and DPPH assays. The radical scavenging activity of empty SLNs, which is below 10% compared to free CUR, shows that the lipid nanoparticles themselves do not contribute to any significant antiradical activity. A significant enhancement in the antioxidative activity of the CUR-loaded SLNs demonstrates the radical scavenging activity of the CUR when loaded within the nanoparticles. Free CUR exhibited a concentration-dependent profile, with increased extract concentrations resulting in improved ABTS antioxidant activity. CUR-loaded SLNs also showed a concentration-dependent profile with a response similar to free CUR (Figure 4A). In fact, the incorporation of CUR within the lipid nanoparticles improves water solubility, thus enhancing the antioxidant activity. At a concentration of 20 μg/mL, the ability to scavenge ABTS*+ of free CUR and CUR-loaded SLNs was ~61.3% and ~55.6%, respectively. Fu and co-workers and Wu et al. [44,45] encapsulated CUR with soy protein isolate and pectin nanocomplexes and self-assembled casein–dextran conjugate micelles, respectively, and similar results appeared in their studies. Furthermore, upon incorporation into SLNs, the antioxidant activity of CUR was similar to its free form between 5 and 20 μg/mL concentrations (Figure 4B). At the concentration of 40 μg/mL, the DPPH radical scavenging activities of free CUR and CUR-loaded SLNs were ~67.5% and ~44.8%, respectively. These results indicated that CUR-loaded SLNs exhibited a lower antioxidant activity than that of free CUR for higher concentrations. This was attributed to the CUR being encapsulated in lipid nanoparticles with a hydrophobic surface, which hamper contact between CUR and free radicals. The DPPH radical scavenging activities of CUR from the present study are in accordance with those of previous studies on the DPPH scavenging activities of [46,47].

### 3.2. Antibacterial Activity of Curcumin and CUR-Loaded SLNs

**Figure 5:**
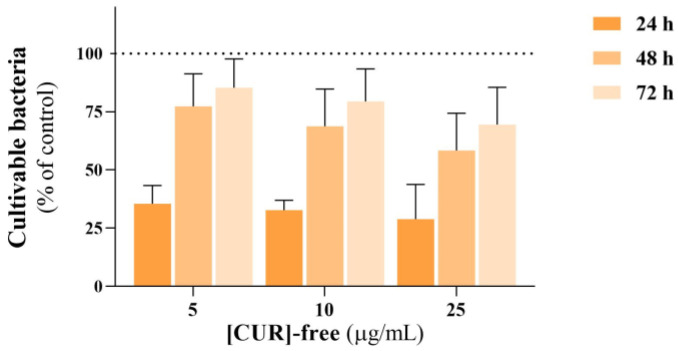
Antibacterial activity of free CUR against *E. faecalis* planktonic growth over a concentration range of 2.5 to 10 µg/mL and up to 72 h of exposure. Control group (dotted line).

Free CUR, at 5 to 25 μg/mL, caused a significant reduction in the CFU counting (around 65%) of planktonic *E. faecalis* after 24 h exposure (Figure 5). However, for longer exposures, i.e., 48 and 72 h, the inhibitory effect was not observed. This might be related to the chemical instability of CUR in the aqueous culture medium, allowing the concomitant growth of the bacterial population [47]. Even without CUR degradation, its concentration may drop to subinhibitory levels that are inadequate to prevent bacterial growth. This may no longer effectively suppress bacterial activity and potentially enable bacterial cells to survive and proliferate [48]. To address this limitation and improve the clinical efficacy of CUR, approaches such as encapsulation in nanoparticles and synergistic combinations with other compounds have been explored [49].

**Figure 6:**
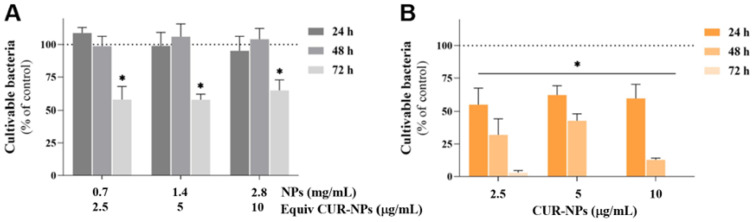
Antibacterial activity of SLNs (**A**) and CUR-loaded SLNs (**B**) against *E. faecalis* planktonic growth over a concentration range of 2.5 to 10 µg/mL in CUR, equivalent to a range of 0.7 to 2.8 mg/mL of empty SLNs, and up to 72 h of exposure. Asterisks (*p* < 0.05) indicate significant differences compared to the control group (dotted line).

The antibacterial activity of SLNs and CUR-loaded SLNs against *E. faecalis* planktonic growth was evaluated over time, and the results are depicted in Figure 6. For SLNs, exposures of 24 and 48 h showed no antibacterial effect. However, a 72 h exposure resulted in a significant reduction in cultivable bacteria by over 50%. In contrast, CUR-loaded SLNs demonstrated a significant, time-dependent impact on the planktonic growth of *E. faecalis*. After just 24 h, the number of cultivable bacteria was reduced to approximately 50% compared to the control group across all concentrations tested. This effect became more pronounced after 48 h of exposure, particularly at 10 µg/mL of CUR-loaded SLNs, which led to a reduction of nearly 70%. The most remarkable results were observed after 72 h of continuous exposure, where a concentration of 2.5 µg/mL reduced the cultivable bacteria to only 3%, and higher concentrations resulted in no detectable cultivable bacteria. While free CUR MIC values against *E. faecalis* range widely depending on time of exposure, CUR-loaded SLNs achieve lower effective doses over extended periods, demonstrating improved antibacterial activity compared to standard CUR formulations [49]. Likewise, Minhaco and co-authors developed CUR-loaded PLGA nanoparticles against the endodontic biofilm [25]. The MIC values for *E. faecalis* were 500 μg/mL for free CUR, while CUR-loaded nanoparticles showed a lower MIC of 200 μg/mL. The current study reveals the potential of CUR-loaded SLNs towards *E. faecalis* over other carriers [25,49]. CUR-loaded SLNs could serve as an effective drug delivery system, enhancing the intracellular concentration of CUR and promoting its accumulation in the mitochondria [50]. Encapsulating CUR in SLNs has been explored as a strategy to improve its delivery, stability, and antimicrobial effectiveness. Although research specifically focusing on CUR-loaded SLNs for *E. faecalis* in endodontics is limited, existing studies highlight several key advantages and mechanisms. SLNs are known to protect CUR from degradation and enhance its bioavailability by embedding it within a solid lipid matrix. This structure not only extends the release of CUR but also helps maintain its activity over time. Tanvi Gupta et al. also observed that the optimized lipid matrix improved the therapeutic index of CUR, reducing toxicity while enhancing efficacy [51].

**Figure 7:**
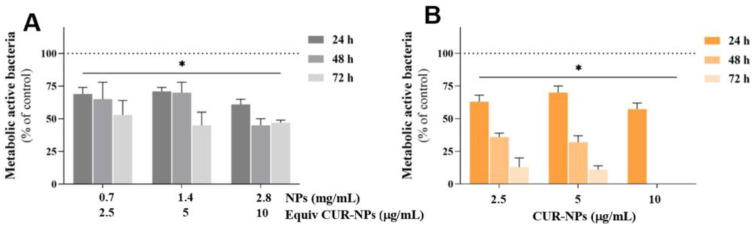
Antibacterial activity of SLNs (**A**) and CUR-loaded SLNs (**B**) against *E. faecalis* biofilm formation over a concentration range of 2.5 to 10 µg/mL in CUR, equivalent to a range of 0.7 to 2.8 mg/mL of empty SLNs, and up to 72 h of exposure. Asterisks (*p* < 0.05) indicate significant differences compared to the control group (dotted line).

Both types of SLN formulations caused a significant reduction in adherent *E. faecalis* across all concentrations and time points tested, as presented in Figure 7.

Specifically, SLNs at concentrations of 2.5 and 5 µg/mL caused a similar reduction, with a mean value of 30% compared to the control group after 24 and 48 h of exposure. Seventy-two hours after SLN addition, this value increased to approximately 50%. At 10 µg/mL, SLNs induced a slightly higher reduction initially, but after 72 h, the reduction plateaued at 50%, similar to the other experimental conditions. In contrast, CUR-loaded SLNs exhibited a clear time-dependent effect. After 24 h, metabolically active bacteria were about 65%, compared to the control group (set at 100%). After 48 h, at concentrations of 2.5 and 5 µg/mL, the metabolic active bacteria were around 35%, further decreasing to 12% after 72 h of exposure. The most significant results were observed at a concentration of 10 µg/mL with longer exposure times, where no metabolically active bacteria were detected after 48 h. Free CUR loses efficacy over time (Figure 5), primarily due to its chemical instability. In aqueous environments, CUR undergoes rapid degradation through processes such as oxidation, hydrolysis, and exposure to light and heat. This instability reduces its bioavailability and antimicrobial activity during prolonged use. However, when CUR is encapsulated within SLNs, its activity is sustained for longer periods compared to free CUR (Figure 6 and Figure 7). The solid lipid core of SLNs protects CUR from environmental factors like light, oxygen, and hydrolysis, significantly slowing down its degradation and preserving its chemical stability. Additionally, SLNs provide controlled and sustained release of CUR, maintaining therapeutic concentrations over extended periods. This controlled release prevents the rapid depletion of CUR levels, ensuring prolonged antimicrobial efficacy against *E. faecalis*. Furthermore, the small size and surface properties of SLNs enhance their penetration into biofilms and infected areas, enabling CUR to act directly at the site of infection. This targeted delivery not only improves efficacy but also minimizes waste and degradation, making SLNs a superior delivery system for maintaining CUR’s therapeutic potential. Sandu and co-workers described the role of CUR-loaded SLNs in mature biofilm disruption in the context of wound healing [52]. Here, the lipid nanoparticles developed extend this effect towards *E. faecalis* in endodontic situations. Moreover, the enhanced depth of penetration and sustained action of your CUR-loaded SLNs make them particularly suitable for endodontic applications. Polymeric nanoparticles of PLGA have demonstrated similar biofilm inhibition but often at higher doses [36]. The present study demonstrates the potential of SLNs to enhance the antimicrobial efficacy of CUR against *E. faecalis*, a pathogen frequently implicated in endodontic infections. However, its in vitro design represents a limitation, as the findings may not fully translate to in vivo conditions, where factors such as host immune response and tissue interactions play a critical role. Additionally, the focus on a single pathogen, while clinically relevant, does not encompass the polymicrobial nature of most endodontic infections. Indeed, multispecies biofilms better reflect clinical conditions, yet they are more challenging to culture and may lead to variability or misinterpreted results. Additionally, no specific combination of species is universally recognized as a better mimic of clinical conditions compared to others. For these reasons, this study focused on a single pathogen to ensure reproducibility and clarity in results. Future research should include in vivo models and a broader spectrum of microbial targets to validate and expand the clinical applicability of these findings.

From the release kinetics study (Figure 3), it is known that CUR is released from the loaded SLNs within several days, which most probably explains the observed time-dependent bacterial inhibition for both planktonic and sessile bacteria. This pattern of antibacterial activity is a most desired feature to eliminate the root canal bacteria that persist after the endodontic treatment [52]. Additionally, the long-term effect was observed at low concentrations, i.e., 2.5 and 5 µg/mL. By contrast, the inhibitory effect of free CUR was observed only for short exposure times, lacking the long-term effect. Polymeric nanoparticles of PLGA have demonstrated similar biofilm inhibition but often at higher doses [36].

### 3.3. Cytocompatibility

**Figure 8:**
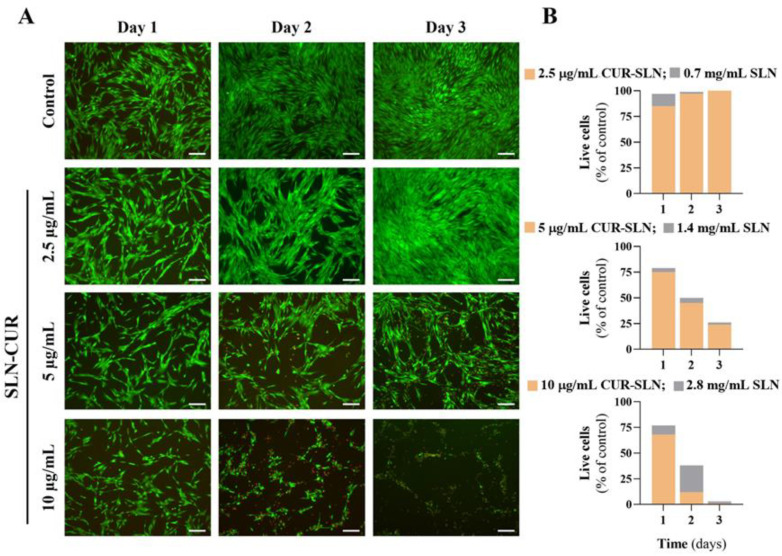
Viability of gingival fibroblasts following 1, 2, and 3 days of exposure to SLNs and CUR-loaded SLNs, assessed using fluorescent-based live/dead staining. (**A**) Representative fluorescent images showing live (green) and dead (red) cells. Scale bar: 200 μm. (**B**) Quantitative analysis of the percentage of live cells relative to the control group (set at 100%).

Cell viability was assessed using a live/dead staining assay after 1, 2, and 3 days of exposure to SLNs and CUR-loaded SLNs. Figure 8A illustrates that in the control group, gingival fibroblasts were well adhered and spread, maintaining their typical elongated morphology.

The cells actively proliferated from day 1 to day 3, with nearly all cells stained green, indicating their viability. In contrast, cells exposed to CUR-loaded SLNs exhibited a clear concentration- and time-dependent decline in both cell number and viability. At 2.5 µg/mL of CUR-loaded SLNs, cells maintained a viability comparable to the control group. However, at 5 µg/mL, a noticeable reduction in cell viability was observed, which became more pronounced over time. The most significant deterioration was observed at the highest concentration tested (10 µg/mL), where almost no viable cells were detected after 72 h of exposure, with a marked increase in dead cells. The quantification of live cells from the collected images enabled a numerical comparison of the viability profiles for both SLN formulations, as shown in Figure 8B. At the lowest concentration tested (2.5 µg/mL), fibroblast viability was similar for both SLN formulations. However, at higher concentrations, a time-dependent decrease in viability was observed, similar for both formulations. Notably, at 10 µg/mL, SLNs maintained higher cell viability compared to CUR-loaded SLNs.

**Figure 9:**
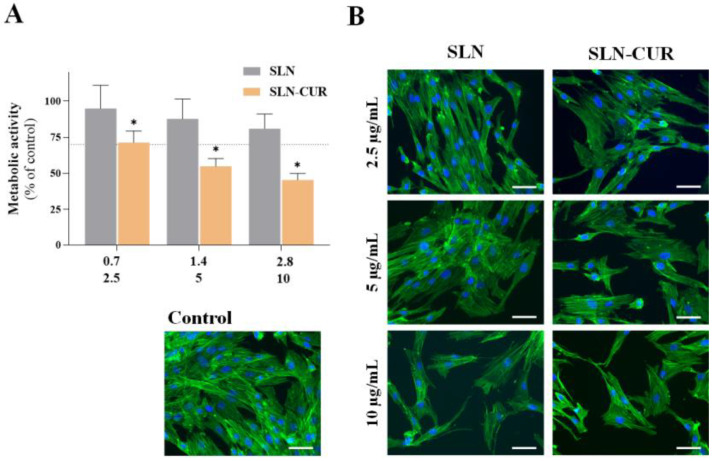
Effect of SLNs and CUR-loaded SLNs on gingival fibroblasts after 24 h of exposure. (**A**) Cell metabolic activity assessed using the MTT assay. The dotted line represents the “cut-off” value of 70% relative to the control. 0.7 to 2.8 mg/mL of NPs and 2.5 to 10 μg/mL of equivalent concentration of CUR-loaded NPs. Asterisks (*p* < 0.05) indicate significant differences compared to the control group (set at 100%). (**B**) Cell morphology evaluated by immunofluorescence staining of the F-actin cytoskeleton (green) and nucleus (blue). Scale bar: 50 µm.

Fibroblast metabolic activity, evaluated by the MTT assay after 24 h of exposure to SLNs and CUR-loaded SLNs, is represented in Figure 9A. Both SLN formulations exhibited a concentration-dependent effect. For SLNs, only the 28 mg/mL equivalent to 10 µg/mL CUR-loaded SLNs caused a significant decrease in cell metabolic activity compared to the control group (set at 100%). Other lipid nanoparticles described by Angelotti and colleagues maintained gingival fibroblast viability at lower doses (22 μg/mL) [53]. In contrast, all tested concentrations of CUR-loaded SLNs adversely affected cell metabolic activity, with values significantly lower than those of the control group. Specifically, at 5 and 10 µg/mL, the percentage of metabolically active cells fell below the “cut-off” value of 70%, indicating potential cytotoxicity.

Regarding F-actin organization, as illustrated in Figure 9B, although the number of cells decreased with increasing concentration, the stained cells displayed well-organized F-actin in parallel linear actomyosin bundles (stress fibers) and prominent nuclei. This suggests that the SLN formulations did not disrupt actin cytoskeleton organization or cell spreading area. Despite the possibility of deleterious cellular effects at concentrations of 5 and 10 µg/mL, it should be noted that this would be relevant only in the rare event of radicular extravasation of the endodontic formulation. Nevertheless, at the low concentration of 2.5 µg/mL, CUR-loaded SLNs are cytocompatible while having long-term activity against *E. faecalis*, fulfilling the clinical desired endodontic purposes of preventing apical periodontitis.

## 4. Conclusions

Curcumin encapsulation in lipid nanoparticles was successfully performed using cetyl palmitate as a solid lipid stabilized by Tween 80 surfactant. The produced lipid nanoparticles exhibited a high CUR encapsulation efficiency (84 ± 8%) and storage stability over 8 weeks at room temperature or 4 °C. The CUR-loaded nanoparticles exhibited excellent water dispersibility, strong antioxidant activity, and adequate physicochemical properties for endodontic application. The antimicrobial activity of CUR was significantly enhanced and sustained over the long term when incorporated into lipid nanoparticles. Additionally, the cytotoxicity of CUR-loaded nanoparticles on gingival fibroblasts proved to be non-cytotoxic, making them a suitable and effective option for intracanal treatments.

While this study highlights the potential of SLNs as carriers for CUR in endodontic therapy, further research is necessary to translate these findings into clinical practice. In vivo studies are critical to evaluate safety, biocompatibility, and therapeutic efficacy in a complex biological environment. Additionally, broader pathogen testing is required to address the polymicrobial nature of endodontic infections. Long-term stability studies should be undertaken to ensure the formulation’s practical applicability, and mechanistic investigations will provide deeper insights into its mode of action.

This study highlights the potential of lipid nanoparticle-based CUR formulations as a safe and efficient intracanal endodontic therapy, paving the way for further investigations into their clinical applications.

## Figures and Tables

**Table 1:** Summary description of mathematical models to understand drug release mechanisms and related calculations.

Mathematical Models and Description	Calculations
Zero-order release model, describes the continuous and constant release of a drug from a drug delivery system over time.	Q = Q_0_ + K_0t_ Q: amount of drug released or dissolved; Q_0_: initial amount of drug in solution (it is usually zero); K_0t_ zero order release constant.
First-order release model, refers to a drug release rate that is proportional to its concentration.	Log C = Log C_0_ − kt/2.303 C_0_: initial concentration of drug; K: first order constant
Hixson–Crowell release model, explains drug release from systems where changes occur in the surface area and particle diameter over time.	Q_0_^1/3^ − Q_t_^1/3^ = KHC_t_ Q_t_: amount of drug released in time t; Q_0_: initial amount of the drug in tablet/formulation; KHC: rate constant for Hixson-Crowell rate equation.
Higuchi release model, relates the drug release rate to physical constants, following the basic principles of diffusion.	Q_t_ = k_H_ (t)^0.5^ Q_t_: amount of drug released in time t; T: time in hours; k_H_: release rate constant for the Higuchi model
Korsmeyer–Peppas release model, characterizes drug release through multiple processes, including diffusion, erosion, and matrix relaxation.	F = M_t_/M N = K_t_n F: fraction of drug release at time t; M_t_/M: fraction of drug released at time; K_t_: the rate constant; n: release exponent.

**Table 2:** Characterization of unloaded and CUR-loaded solid lipid nanoparticles.

Formulation	Size (nm)	PDI	ζ-Potential (mV)	EE (%)
SLN	208 ± 7	0.158 ± 0.006	−25 ± 2	
CUR-loaded SLN	206 ± 6	0.155 ± 0.038	−26 ± 6	84 ± 8

## Data Availability

Data are contained within the article.

## References

[1] Waltimo T., Trope M., Haapasalo M., Ørstavik D. (2005). Clinical Efficacy of Treatment Procedures in Endodontic Infection Control and One Year Follow-Up of Periapical Healing. J. Endod..

[2] Paqué F., Balmer M., Attin T., Peters O.A. (2010). Preparation of Oval-shaped Root Canals in Mandibular Molars Using Nickel-Titanium Rotary Instruments: A Micro-computed Tomography Study. J. Endod..

[3] Orstavik D., Haapasalo M. (1990). Disinfection by endodontic irrigants and dressings of experimentally infected dentinal tubules. Dent. Traumatol..

[4] Peters O.A., Schönenberger K., Laib A. (2001). Effects of four Ni-Ti preparation techniques on root canal geometry assessed by micro computed tomography: Canal preparation assessed by micro-CT. Int. Endod. J..

[5] Desai S., Chandler N. (2009). Calcium Hydroxide–Based Root Canal Sealers: A Review. J. Endod..

[6] Best S., Ammons C.L., Karunanayake G.A., Saemundsson S.R., Tawil P.Z. (2021). Outcome Assessment of Teeth with Necrotic Pulps and Apical Periodontitis Treated with Long-term Calcium Hydroxide. J. Endod..

[7] Siqueira J.F., Lopes H.P. (1999). Mechanisms of antimicrobial activity of calcium hydroxide: A critical review. Int. Endod. J..

[8] Haapasalo H.K., Sirén E.K., Waltimo T.M.T., Òrstavik D., Haapasalo M.P.P. (2000). Inactivation of local root canal medicaments by dentine: An in vitro study. Int. Endod. J..

[9] Portenier I., Haapasalo H., Rye A., Waltimo T., Ørstavik D., Haapasalo M. (2001). Inactivation of root canal medicaments by dentine, hydroxylapatite and bovine serum albumin. Int. Endod. J..

[10] Yamamoto L.Y., Loureiro C., Cintra L.T.A., Leonardo R.d.T., Banci H.A., Ribeiro A.P.F., Sivieri-Araujo G., Jacinto R.d.C. (2021). Antibiofilm activity of laser ablation with indocyanine green activated by different power laser para. Antibiofilm activity of laser ablation with indocyanine green activated by different power laser parameters compared with photodynamic therapy on root canals infected with *Enterococcus faecalis*. Photodiagnosis Photodyn. Ther..

[11] Barbosa-Ribeiro M., De-Jesus-Soares A., Zaia A.A., Ferraz C.C.R., Almeida J.F.A., Gomes B.P.F.A. (2016). Antimicrobial Susceptibility and Characterization of Virulence Genes of Enterococcus faecalis Isolates from Teeth with Failure of the Endodontic Treatment. J. Endod..

[12] Ghorbanzadeh R., Assadian H., Chiniforush N., Parker S., Pourakbari B., Ehsani B., Alikhani M.Y., Bahador A. (2020). Modulation of virulence in Enterococcus faecalis cells surviving antimicrobial photodynamic inactivation with reduced graphene oxide-curcumin: An ex vivo biofilm model. Photodiagnosis Photodyn. Ther..

[13] Righeschi C., Bergonzi M.C., Isacchi B., Bazzicalupi C., Gratteri P., Bilia A.R. (2016). Enhanced curcumin permeability by SLN formulation: The PAMPA approach. LWT Food Sci. Technol. Int..

[14] Picco D.D.C.R., Cavalcante L.L.R., Trevisan R.L.B., Souza-Gabriel A.E., Borsatto M.C., Corona S.A.M. (2019). Effect of curcumin-mediated photodynamic therapy on *Streptococcus mutans* and *Candida albicans*: A systematic review of in vitro studies. Photodiagnosis Photodyn. Ther..

[15] Vasiliu S., Racovita S., Gugoasa I.A., Lungan M.A., Popa M., Desbrieres J. (2021). The Benefits of Smart Nanoparticles in Dental Applications. Int. J. Mol. Sci..

[16] Rai M., Ingle A.P., Pandit R., Paralikar P., Anasane N., Santos C.A.D. (2020). Curcumin and curcumin-loaded nanoparticles: Antipathogenic and antiparasitic activities. Expert. Rev. Anti Infect. Ther..

[17] Araújo N.C., de Menezes R.F., Carneiro V.S.M., dos Santos-Neto A.P., Fontana C.R., Bagnato V.S., Harvey C.M., Gerbi M.E.M. (2017). Photodynamic Inactivation of Cariogenic Pathogens Using Curcumin as Photosensitizer. Photobiomodul. Photomed. Laser Surg..

[18] Adamczak A., Ożarowski M., Karpiński T.M. (2020). Curcumin, a Natural Antimicrobial Agent with Strain-Specific Activity. Pharmaceuticals.

[19] Song J., Choi B., Jin E.J., Yoon Y., Choi K.H. (2012). Curcumin suppresses *Streptococcus mutans* adherence to human tooth surfaces and extracellular matrix proteins. Eur. J. Clin. Microbiol. Infect. Dis..

[20] Shahverdi A., Moghaddam K., Iranshahi M., Yazdi M. (2009). The combination effect of curcumin with different antibiotics against Staphylococcus aureus. Int. J. Green Pharm..

[21] Tyagi P., Singh M., Kumari H., Kumari A., Mukhopadhyay K. (2015). Bactericidal Activity of Curcumin I Is Associated with Damaging of Bacterial Membrane. PLoS ONE.

[22] Anand P., Kunnumakkara A.B., Newman R.A., Aggarwal B.B. (2007). Bioavailability of Curcumin: Problems and Promises. Mol. Pharm..

[23] Slika L., Patra D. (2020). A short review on chemical properties, stability and nano-technological advances for curcumin delivery. Expert. Opin. Drug Deliv..

[24] Thambirajoo M., Maarof M., Lokanathan Y., Katas H., Ghazalli N.F., Tabata Y., Fauzi M.B. (2021). Potential of Nanoparticles Integrated with Antibacterial Properties in Preventing Biofilm and Antibiotic Resistance. Antibiotics.

[25] Minhaco V.M.T.R., Huacho P.M.M., Imbriani M.J.M., Tonon C.C., Chorilli M., de Souza Rastelli A.N., Spolidorio D.M.P. (2023). Improving antimicrobial activity against endodontic biofilm after exposure to blue light-activated novel curcumin nanoparticle. Photodiagnosis Photodyn. Ther..

[26] Pourhajibagher M., Plotino G., Chiniforush N., Bahador A. (2020). Dual wavelength irradiation antimicrobial photodynamic therapy using indocyanine green and metformin doped with nano-curcumin as an efficient adjunctive endodontic treatment modality. Photodiagnosis Photodyn. Ther..

[27] Yasini Z., Roghanizad N., Fazlyab M., Pourhajibagher M. (2022). Ex vivo efficacy of sonodynamic antimicrobial chemotherapy for inhibition of *Enterococcus faecalis* and *Candida albicans* biofilm. Photodiagnosis Photodyn. Ther..

[28] Ipar V.S., Dsouza A., Devarajan P.V. (2019). Enhancing Curcumin Oral Bioavailability Through Nanoformulations. Eur. J. Drug Metab. Pharmacokinet..

[29] Mierzejewska Ż.A., Rusztyn B., Łukaszuk K., Borys J., Borowska M., Antonowicz B. (2024). The Latest Advances in the Use of Nanoparticles in Endodontics. Appl. Sci..

[30] Omidian H., Wilson R.L., Chowdhury S.D. (2023). Enhancing Therapeutic Efficacy of Curcumin: Advances in Delivery Systems and Clinical Applications. Gels.

[31] Chaubal T.V., Ywen B.S., Ying Ying T., Bapat R. (2024). Clinical and microbiologic effect of local application of curcumin as an adjunct to scaling and root planing in periodontitis: Systematic review. Ir. J. Med. Sci..

[32] Bapat R.A., Bedia S.V., Bedia A.S., Yang H.J., Dharmadhikari S., Abdulla A.M., Chaubal T.V., Bapat P.R., Abullais S.S., Wahab S. (2023). Current appraises of therapeutic applications of nanocurcumin: A novel drug delivery approach for biomaterials in dentistry. Environ. Res..

[33] Rahmani A.H., Alsahli M.A., Aly S.M., Khan M.A., Aldebasi Y.H. (2018). Role of Curcumin in Disease Prevention and Treatment. Adv. Biomed. Res..

[34] Borges A., De Freitas V., Mateus N., Fernandes I., Oliveira J. (2020). Solid Lipid Nanoparticles as Carriers of Natural Phenolic Compounds. Antioxidants.

[35] Mirchandani Y., Patravale V.B., Brijesh S. (2021). Solid lipid nanoparticles for hydrophilic drugs. J. Control. Release.

[36] Ferreira M., Silva E., Barreiros L., Segundo M.A., Costa Lima S.A., Reis S. (2016). Methotrexate loaded lipid nanoparticles for topical management of skin-related diseases: Design, characterization and skin permeation potential. Int. J. Pharm..

[37] Elmsmari F., González Sánchez J.A., Duran-Sindreu F., Belkadi R., Espina M., García M.L., Sánchez-López E. (2021). Calcium hydroxide-loaded PLGA biodegradable nanoparticles as an intracanal medicament. Int. Endod. J..

[38] Shah R., Eldridge D., Palombo E., Harding I. (2015). Lipid Nanoparticles: Production, Characterization and Stability.

[39] Kurien B.T., Singh A., Matsumoto H., Scofield R.H. (2007). Improving the solubility and pharmacological efficacy of curcumin by heat treatment. Drug Dev. Technol..

[40] Yingchoncharoen P., Kalinowski D.S., Richardson D.R. (2016). Lipid-Based Drug Delivery Systems in Cancer Therapy: What Is Available and What Is Yet to Come. Pharmacol. Rev..

[41] Yeo S., Kim M.J., Shim Y.K., Yoon I., Lee W.K. (2022). Solid Lipid Nanoparticles of Curcumin Designed for Enhanced Bioavailability and Anticancer Efficiency. ACS Omega.

[42] Silva A.C., Kumar A., Wild W., Ferreira D., Santos D., Forbes B. (2012). Long-term stability, biocompatibility and oral delivery potential of risperidone-loaded solid lipid nanoparticles. Int. J. Pharm..

[43] Slavova-Kazakova A., Janiak M.A., Sulewska K., Kancheva V.D., Karamać M. (2021). Synergistic, additive, and antagonistic antioxidant effects in the mixtures of curcumin with (−)-epicatechin and with a green tea fraction containing (−)-epicatechin. Food Chem..

[44] Fu L., Tan S., Si R., Qiang Y., Wei H., Huang B., Shi M., Fang L., Fu J., Zeng S. (2023). Characterization, stability and antioxidant activity of curcumin nanocomplexes with soy protein isolate and pectin. Curr. Res. Food Sci..

[45] Wu Y., Wang X. (2017). Binding, stability, and antioxidant activity of curcumin with self-assembled casein–dextran conjugate micelles. Int. J. Food Prop..

[46] Waiprib Y., Ingrungruengluet P., Worawattanamateekul W. (2023). Nanoparticles Based on Chondroitin Sulfate from Tuna Heads and Chitooligosaccharides for Enhanced Water Solubility and Sustained Release of Curcumin. Polymers.

[47] Wu J., Chen J., Wei Z., Zhu P., Li B., Qing Q., Chen H., Lin W., Lin J., Hong X. (2023). Fabrication, Evaluation, and Antioxidant Properties of Carrier-Free Curcumin Nanoparticles. Molecules.

[48] Kharat M., Du Z., Zhang G., McClements D.J. (2017). Physical and Chemical Stability of Curcumin in Aqueous Solutions and Emulsions: Impact of pH, Temperature, and Molecular Environment. J. Agric. Food Chem..

[49] Hussain Y., Alam W., Ullah H., Dacrema M., Daglia M., Khan H., Arciola C.R. (2022). Antimicrobial Potential of Curcumin: Therapeutic Potential and Challenges to Clinical Applications. Antibiotics.

[50] Jiang S., Zhu R., He X., Wang J., Wang M., Qian Y., Wang S. (2016). Enhanced photocytotoxicity of curcumin delivered by solid lipid nanoparticles. Int. J. Nanomed..

[51] Gupta T., Singh J., Kaur S., Sandhu S., Singh G., Kaur I.P. (2020). Enhancing Bioavailability and Stability of Curcumin Using Solid Lipid Nanoparticles (CLEN): A Covenant for Its Effectiveness. Front. Bioeng. Biotechnol..

[52] Sandhu S.K., Kumar S., Raut J., Singh M., Kaur S., Sharma G., Roldan T.L., Trehan S., Holloway J., Wahler G. (2021). Systematic Development and Characterization of Novel, High Drug-Loaded, Photostable, Curcumin Solid Lipid Nanoparticle Hydrogel for Wound Healing. Antioxidants.

[53] Angellotti G., Di Prima G., D’Agostino F., Peri E., Tricoli M.R., Belfiore E., Allegra M., Cancemi P., De Caro V. (2023). Multicomponent Antibiofilm Lipid Nanoparticles as Novel Platform to Ameliorate Resveratrol Properties: Preliminary Outcomes on Fibroblast Proliferation and Migration. Int. J. Mol. Sci..

